# Nucleation Kinetics Reveals a Distinct Biological Function Space of Biomolecular Condensates

**DOI:** 10.1002/advs.202522585

**Published:** 2026-04-07

**Authors:** Leif‐Thore Deck, Sandra Navarro Pacheco, Hannes Ausserwöger, Daan Frenkel, Nadia A. Erkamp, Tuomas P. J. Knowles

**Affiliations:** ^1^ Yusuf Hamied Department of Chemistry University of Cambridge Cambridge UK; ^2^ Institute for Complex Molecular Systems Eindhoven University of Technology Eindhoven The Netherlands

**Keywords:** interfaces, nucleation, phase separation, soft matter, thermodynamics

## Abstract

The control of the assembly, size, and surface–volume ratio of biomolecular condensates is crucial to their function in living cells. These features are determined by nucleation from a supersaturated solution, but quantitative measurements of nucleation rates and their comparison across biomolecular systems have been challenging to obtain. Using microfluidics, we measure the nucleation rates of dense liquid phases within dilute solutions and the rates of the reverse process, in which dilute voids form inside condensates. We find that both processes are controlled by a single physical parameter, the interfacial tension between the dense and dilute phases, demonstrating that nucleation in both directions proceeds through essentially a single‐step mechanism. Furthermore, analysis of interfacial tension data establishes rapid nucleation as a general feature of condensates, even if the free‐energy gain per molecule is smaller than the thermal energy. This behavior sets condensates apart from solid crystals or aggregates and highlights the unique physical principles underlying their distinct biological functions.

## Graphical abstract

This study utilizes microfluidics to quantify the nucleation rates of dense liquid phases within dilute solutions and of the reverse process, in which dilute voids nucleate inside condensates. The interfacial tension is identified as the key determinant of both processes. Notably, the analysis reveals that condensates nucleate rapidly even when the free‐energy gain per molecule is smaller than the thermal energy. This behavior sets condensates apart from solid crystals or aggregates and highlights the unique physical principles underlying their distinct biological functions.

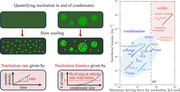

## 1 Introduction

**FIGURE 1:**
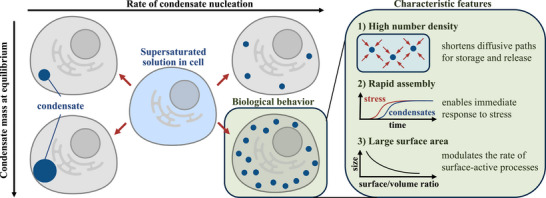
Nucleation as determinant of condensate function. While thermodynamics modulates the mass and volume of condensates at equilibrium, kinetics governs the rate of assembly as well as the number and size distribution of condensates. Only if nucleation is rapid, large numbers of condensates can form quickly and provide an effective stress response.

Numerous biomolecules, including proteins, peptides, and nucleic acids can phase separate both in vivo and in vitro [1, 2, 3]. During phase separation, spontaneous demixing of an initially homogeneous solution occurs, resulting in a phase depleted in the biomolecule, and one enriched in it: the condensate [4, 5]. Condensates have been shown to modulate numerous biological processes [5, 6, 7, 8, 9] and there is growing evidence of their involvement in a range of human diseases [10, 11]. While substantial progress has been made in characterizing the thermodynamics of phase separation [12, 13, 14], the study of the fundamental molecular events that govern the associated kinetics, including nucleation, growth and coalescence rates of condensates, has been more challenging. This is because condensates are nanometer‐sized when they nucleate and undergo significant growth and coalescence before detection: the early stages of nucleation cannot be observed directly and the number of condensates observed later has an indirect relationship with the rate of nucleation [15, 16, 17, 18]. Condensate nucleation governs the dynamics through which a living cell responds to stress [15, 19, 20], regulates the number, size, and surface–volume ratio of condensates [21, 22], and modulates the rate of processes that take place at condensate interfaces [9, 23, 24, 25]. Its quantification therefore promises to deepen the understanding of the biological functions of condensates, as outlined in Figure 1.

Here, we introduce an approach to quantify the nucleation kinetics. We combine measurements of the nucleation of condensates within a dilute solution with measurements of the inverse process, where dilute phase voids are formed within condensates. We achieve accurate determination of individual nucleation events by measuring this process in small volumes using microfluidics. This method bypasses the limitations of conventional assays and involves characterizing four physical aspects: the thermodynamics of phase separation, diffusion within the condensates, condensate shrinkage due to solvent expulsion, and nucleation of voids within the condensates. Our analysis shows that both the nucleation of dense phases within dilute solutions and the nucleation of dilute voids within dense condensates are governed by a single physical parameter, the interfacial tension between the two phases, indicating that these processes proceed through a single‐step mechanism. Building on Classical Nucleation Theory (CNT) [26, 27, 28, 29], we explore how to infer nucleation kinetics for condensate formation under a variety of conditions. This motivates a broad analysis of interfacial tension data and reveals rapid nucleation as a general feature of biomolecular condensates that provides access to a biological function space distinct from that of solid aggregates or crystals: rapid nucleation is a prerequisite both for effective stress response and for all biological functions that require high number densities and large surface areas of condensates.

## 2 Results

### 2.1 Thermodynamic Driving Force for the Formation of Voids in Condensates

**FIGURE 2:**
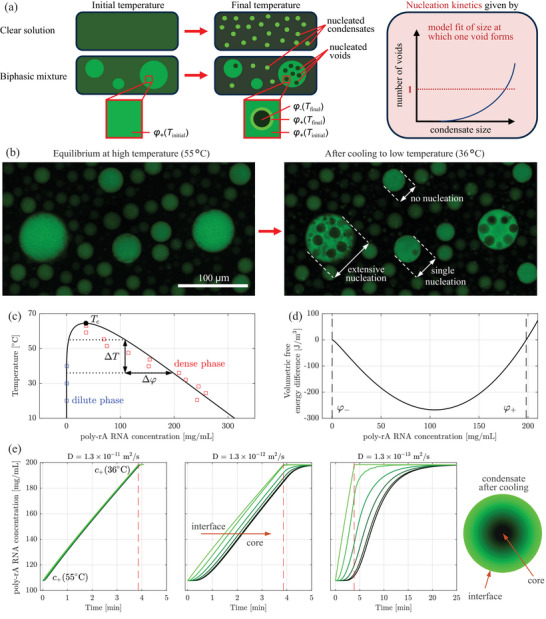
Driving force of temperature‐induced phase separation. (a) Schematic of the nucleation phenomena that take place when cooling either a clear or a phase‐separated solution of poly‐rA RNA. (b) Confocal images taken before and after cooling from 55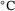 to 36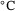. Voids nucleate in sufficiently large condensates and small condensates nucleate in the dilute phase. (c) Binodal measurements of dense (red markers) and dilute phases (blue markers) and Flory‐Huggins fit (black line). The increase in equilibrium concentration of the dense phase upon cooling drives the nucleation of voids. (d) Free energy difference between mixed and phase separated states as a function of poly‐rA RNA concentration at 40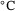. (e) Simulations of the concentration evolution in a condensates with r=20μm upon cooling from 55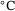 to 36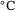 for three different diffusion coefficients. The experimental value is shown in the right panel, where diffusion is sufficiently slow that major concentration gradients between condensate core (black) and the dense–dilute interface (green) emerge.

We first explore the nucleation of voids, that is, of pockets of dilute phase within the dense phase of condensates. We use a model system [30, 31] consisting of poly‐rA RNA (700–3500 kDa) in 1M KCl (see Section 4.1). As shown in Figure 2a,b, this system forms spherical condensates at elevated temperature with physical properties (low diffusivity of about $10^{-13}\;\;m^{2}\;s^{-1}$, low dense phase concentration of <300 mg mL^−1^, proximity to criticality) that are representative of biomolecular condensates [5]. Upon cooling, a large number of voids form in large condensates, whereby the number of voids scales with the size of the condensate. Voids behave dynamically: they nucleate, grow, and fuse, both with each other and with the surrounding dilute phase (see Figure S5).

These phenomena take place because the binodal curve that describes the composition of the coexisting dense and dilute phases widens upon cooling (see Figure 2c). NanoDrop measurements of samples incubated and spun down at the specified temperature provided dilute phase concentrations (see Methods). Dense phase concentrations were estimated from photon count intensities measured on a Leica Stellaris 5 confocal microscope (see Methods). The phase coexistence can be described using a mean field (Flory‐Huggins) model of a polymer in solvent, taking into account the translational entropy of the solute and solvent and their binary interactions in the dense and dilute phases, yielding an expression of the form: $f/\left(k_{B}T\right)=\varphi \ln\left(\varphi \right)/M+\left(1-\varphi \right)\ln\left(1-\varphi \right)+\varphi \left(1-\varphi \right)\chi \left(T\right)$ where $k_{B}$ is the Boltzmann constant, φ the volume fraction of the solute (i.e., of RNA), M its effective chain length, and χ(T) the Flory interaction parameter [32, 33, 34, 35]. Volume fractions were converted into mass concentrations assuming an RNA density of 1.6 g mL^−1^ [36]. For each given set of parameters, the binodal concentrations were computed using the convex hull algorithm [34, 37], a recent numerical implementation of the graphical construction method originally proposed by Gibbs [38, 39]. Figure 2c shows the simulated binodals obtained from fitting the interaction parameter to the experimental data (see Section 4.2), revealing a critical temperature of 64.5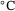.

Of particular interest is the free‐energy barrier that must be overcome to form a nucleus of the new phase. Following Classical Nucleation Theory (CNT) [26, 27, 28] (see refs. [40, 41] for more recent reviews), the free energy required to form a dilute phase cluster comprising n solute molecules is

(1) $$W_{-}\left(n\right)=\gamma A\left(n\right)+n\Delta g_{-}$$

where A(n) denotes the surface area of the cluster that scales with $n^{2/3}$, γ the interfacial tension between the dense and dilute phases, and $\Delta g_{-}$ the difference in free energy per solute molecule between the two phases. To compute $\Delta g_{-}$, we first rescale the free energy from a per unit volume basis in the Flory‐Huggins model to a per molecule basis, as to g=fM/φ. We then compare the free energy of a control volume (see the zoom‐in in Figure 2a) made up of the initial dense phase with that of a two‐phase system comprising both the void and concentrated dense phase

(2) $$\Delta g_{-}=\left(g\left(\varphi_{-}\right)-g\left(\varphi \right)\right)+K\left(\varphi ,\varphi_{\pm }\right)\left(g\left(\varphi_{+}\right)-g\left(\varphi \right)\right)$$

where $\varphi_{\pm }$ denotes the binodal concentrations of dense (+) and dilute (‐) phase, respectively. The first term describes the free‐energy change associated with the transition of an RNA molecule and its solvent from the initial dense phase to the dilute void phase.

The second term results from the exclusion of RNA molecules from the void, which increases their concentration in the surrounding dense phase, where the partition factor K is obtained from a volume balance (see Section 4.3). Figure 2d reports the volumetric free energy difference $\Delta g_{-}/v_{m,-}$ as a function of RNA volume fraction at 40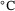. $\Delta g_{-}$ equals zero at the two binodal concentrations, as it should be, as there is no driving force for nucleation when the system is in equilibrium. For $\varphi_{-}<\varphi <\varphi_{+}$, $\Delta g_{-}<0$ holds, implying that phase separation is thermodynamically favorable. With increasing distance from the dense phase binodal, the driving force becomes more negative, again in line with expectations, before it eventually reaches a minimum and approaches zero at the dilute phase binodal.

Considering again Equation (1), we find that $\Delta g_{-}$ is negative, while the interfacial tension γ is positive. Hence, $W_{-}$ must be positive for sufficiently small clusters (values of n), before it experiences a maximum for intermediate sizes, and eventually becomes negative for larger ones. Its maximum value constitutes the work required to form a critical nucleus of size $n_{c}$, which is computed as $W_{c,-}=16\pi \gamma^{3}v_{m,-}^{2}/\left(3\Delta g_{-}^{2}\right)$. $v_{m,\pm }=M_{w}/\left(N_{A}{\hat{c}}_{\pm }\right)$ denotes the average volume of dilute or dense phase in which one solute molecule resides, which is linked to the binodal mass concentration $\hat{c}$ through the Avogadro constant $N_{A}$ and the solute's molecular weight $M_{w}$. Note that while the RNA used in the experiments is polydisperse, for the sake of simplicity, a single value of $M_{w}=2500$ kg mol^−1^ is used in the calculations. The nucleation rate $J_{\mathrm{void}}$, which denotes the expected number of nucleation events per unit time and per unit volume, is given as

(3) $$J_{\mathrm{void}}=\vartheta D_{+}c\sqrt{\frac{\Delta g_{-}^{2}}{v_{m,-}^{2}\gamma k_{b}T}}\exp\left(-\frac{W_{c,-}}{k_{b}T}\right)$$

where $D_{+}$ is the diffusion coefficient of the RNA and ϑ a dimensionless correction factor. The detailed derivation of Equation (3) is provided in Section 4.3. As J depends on RNA concentration, any quantitative description of nucleation requires knowledge of the time‐dependent concentration field in the condensate that arises from diffusive mass transfer upon cooling. To this end, we proceed by measuring the diffusion coefficient $D_{+}$ and introducing a mechanistic mass transfer model.

### 2.2 Slow Diffusion Generates Out‐of‐Equilibrium Systems

Nuclei require a sufficiently large driving force to form, which means that the RNA concentration in the condensate must deviate appreciably from its equilibrium value. Cooling may induce such a driving force because the binodal concentrations depend on temperature, as shown in Figure 2c: when cooling a dense phase that is initially in equilibrium with the dilute phase, it will no longer be in equilibrium with the dilute phase if there is no time for the RNA concentration to adjust to its new equilibrium value.

To increase its RNA concentration, a condensate may collect additional solute molecules from the surrounding dilute phase or expel solvent. Solvent expulsion is expected to be the main mechanism, because the RNA concentration in the dense phase is 2−3 orders of magnitude higher than in the dilute phase; this suggests that the diffusion‐limited RNA collection from the dilute phase will be relatively slow and of negligible extent. At the dense–dilute interface, the concentration of the condensate rapidly equilibrates by ejecting solvent molecules into the dilute phase; therefore, the RNA concentration on the condensate side of the interface will rapidly approach the new binodal value. To equilibrate the entire volume of the condensate, the RNA molecules must diffuse away from the interface to the core of the condensate, which results in condensate shrinkage. Inward diffusion of the RNA is accompanied by outward diffusion of the solvent to prevent pressure buildup in the condensate. This outward diffusion is unlikely to contribute to the overall dynamics of equilibration because water molecules are substantially smaller than RNA molecules and thus diffuse faster. This is even more pronounced in condensates for which viscosity has been shown to be size‐dependent, resulting in rapid dynamics of small molecules within the polymer scaffold [42].

However, because RNA diffusion is slow, there will initially be a significant driving force for nucleation of voids in the bulk of the condensate. We tested this hypothesis through both experimental measurements of the diffusion coefficient using fluorescence recovery after photobleaching (FRAP) and mechanistic modeling of diffusion in the condensate. FRAP measurements (see Section 4.1 and Figure S3 for details) revealed a value of $D_{+}=1.3\times 10^{-13}\;\;m^{2}\;s^{-1}$ at a temperature of 45.6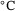. For a condensate with a radius r of 20 μm, the characteristic time for concentration equilibration within the condensate is of the order $r^{2}/\left(6D\right)\approx 10$ min. The value of $D_{+}$ matches literature values [42, 43] of protein‐ and RNA‐based condensates that are routinely found to be on the order of $10^{-12}-10^{-13}\;\;m^{2}\;s^{-1}$; due to the large molecular size of the poly‐rA RNA used here, we expect a diffusion coefficient on the lower end of this range, in line with the measurements.

We proceed by quantifying the concentration gradients that form upon cooling through numerical simulations using a finite difference method. In these simulations, the condensate interface remains in equilibrium with the dilute phase upon cooling, so that the concentration at the interface increases in line with the binodal concentration. The amount of RNA contained in the condensate is imposed to remain constant over time: the condensate shrinks as it becomes more dense. The model is explained in more detail in Section 4.4. The simulations shown in Figure 2e confirm that slow diffusion facilitates the formation of relevant concentration gradients within a condensate of a typical size (radius of 20 μm) that is cooled at a rate of 5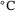 min^−1^ from a temperature of 55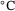 to 36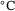 and held at 36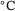 until equilibrium is reached. If diffusion is sufficiently fast (e.g., for $D_{+}=1.3\times 10^{-11}\;\;m^{2}\;s^{-1}$), the concentration evolves uniformly within the condensate, and there are no relevant differences between the dense–dilute interface (green) and the core (black). In the case of slower diffusion, concentration gradients emerge upon cooling, and for the experimental value of $D_{+}=1.3\times 10^{-13}\;\;m^{2}\;s^{-1}$, more than ten minutes are required for the condensate to equilibrate. During this time, the condensate experiences a driving force that may enable void nucleation, as discussed below.

### 2.3 Small Interfacial Tensions Promote the Nucleation of Voids

When quantifying the nucleation kinetics of voids, condensates that form exactly one void throughout the cooling process are of particular interest. If multiple voids nucleate, each void affects the nucleation of further voids because their existence influences the concentration field in the condensate; accurately accounting for this interdependence between voids and the concentration field would go beyond currently available modeling capabilities.

**FIGURE 3:**
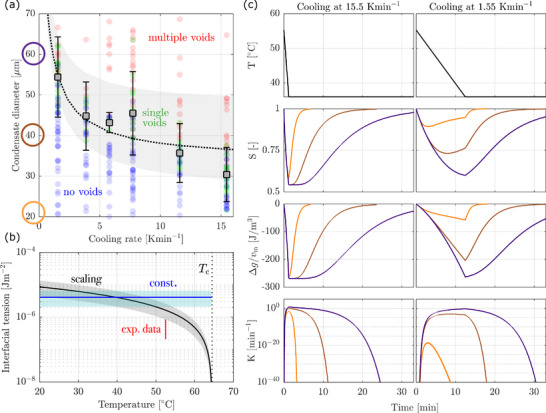
Quantifying the nucleation of voids in condensates. (a) Measurement of the number of voids that form in condensates of different sizes at multiple cooling rates. Each circle represents a condensate in which zero (blue), one (green), or multiple (red) condensates have formed. The square markers indicate the critical size at which in average one void forms and the error bars denote two standard deviations. The black dashed line reports the best fit of the critical sizes and its uncertainty (shaded region). (b) Interfacial tension as a function of temperature. The black line represents the fit using the Ising temperature‐scaling and the blue line the fit assuming a temperature‐independent γ, with uncertainties given as shaded regions. The red line indicates the experimental value from the condensate fusion experiments. (c) Simulations of three condensates (20, 40, 60 μm in diameter, colors) at two cooling rates (columns). The rows indicate the evolution of temperature, of saturation ratio and volumetric free energy difference both in the core of the condensate, and of the nucleation frequency, i.e., the number of voids formed per unit time.

For this reason, we analyzed the critical size of condensates at which a single void forms as a function of the cooling rate. This is shown in Figure 3a: each marker represents a condensate and its color indicates whether zero (blue), one (green), or multiple (red) voids formed. At all cooling rates, larger condensates are found to form more voids. With increasing cooling rate, the critical size (squares) decreases. This is expected because both fast cooling and large condensate size make it more difficult for diffusion to act against the formation of concentration gradients that drive nucleation. We further note that the size regions with zero, one, and multiple voids overlap with each other. This is due to the stochastic nature of nucleation, which implies that the number of voids formed is a random variable [44]. To account for this, we monitored a large number of condensates per cooling rate and computed the critical size as the arithmetic mean size of the condensates that formed one void.

Conceptually, the expected number of voids, N, that form within a condensate is obtained by integrating the nucleation rate given by Equation (3) over the condensate's volume and the duration of the process:

(4) $$N=\int_{t_{0}}^{t_{\mathrm{end}}}K\left(t\right)dt=\int_{t_{0}}^{t_{\mathrm{end}}}\int_{0}^{R(t)}4\pi r^{2}J\left(c\left(r,t\right),T\left(t\right)\right)\;dr\;dt$$

where the time at which the process is completed, $t_{\mathrm{end}}$, is the time when diffusion has leveled out the concentration gradients. The expression for J contains two parameters that are to be estimated from experimental data, namely the interfacial tension γ and the pre‐factor ϑ. They are obtained by fitting the experimental critical sizes, $R_{0,k}$ as a function of the cooling rate k, to simulations, as explained in detail in Section 4.4. As shown in Figure 3a, the simulations describe the experimental critical sizes well: the black dashed line reports the best fit and the shaded region indicates the size range in which condensates are expected to form one void. This range was computed considering that nucleation is a Poisson process for which N given by Equation (4) represents the expected value (see Section S.1) [44, 45, 46]. Interestingly, the error bars of the experimental critical sizes (±2 standard deviations) are of similar magnitude as the confidence interval of the simulations, which indicates that the stochastic nature of nucleation represents the main source of variability in the experimental data. In addition, the best fit of the critical sizes lies within the error bars of the experimental data at all cooling rates. This supports the notion that the model accurately describes the underlying physical phenomena of phase separation coupled with nucleation.

To further probe the accuracy of the model predictions, we evaluated the best‐fit values for the interfacial tension. We first parameterized the interfacial tension using a scaling law derived from thermodynamic considerations assuming the Ising universality class [47, 48], resulting in $\gamma \left(T\right)=\gamma_{0}{\left(\frac{T_{c}-T}{T_{c}}\right)}^{1.26}$. This relation has been used in the simulations shown in Figure 3a that accurately fit the critical sizes, indicating that it describes well the experimental nucleation behavior. We proceeded by testing a modified version of this scaling law [48, 49] by integrating the Ising scaling of the binodal concentrations with temperature, resulting in $\gamma \left(T\right)=\gamma_{0}{\left(\varphi_{+}\left(T\right)-\varphi_{-}\left(T\right)\right)}^{3.9}$ and observed a significantly worse fit of the experimental critical sizes (see Figure S2). This i that the Ising scaling does not accurately describe the temperature‐dependence of the binodal concentrations and that the parameter estimation approach allows for model discrimination. We further tested the simple case of a temperature‐independent γ, which surprisingly resulted in a close fit similar to that obtained through the Ising temperature‐scaling. This is because parameters are estimated for condensates in which one void nucleates: this void forms at or close to the final temperature (36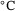) so that the information extracted through this approach corresponds to the interfacial tension at temperatures close to this value. Figure 3b reports the interfacial tension obtained through the Ising temperature‐scaling in the range of 20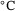 to 65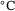 as well as the temperature‐independent value. The associated uncertainties (shaded regions) were obtained by propagating the uncertainty of the thermodynamic parameters resulting from the fit to the Flory‐Huggins model (see Section 4.2). As expected, the interfacial tension predicted by the scaling law decreases with temperature and equals the constant value at $T=39^{\circ }C$, close to the minimum temperature in the experiment. The values obtained on the order of a few $\;\mu J\;m^{-2}$ match those reported in a wide body of literature for other condensate systems, as shown in Figure 5a.

**FIGURE 4:**
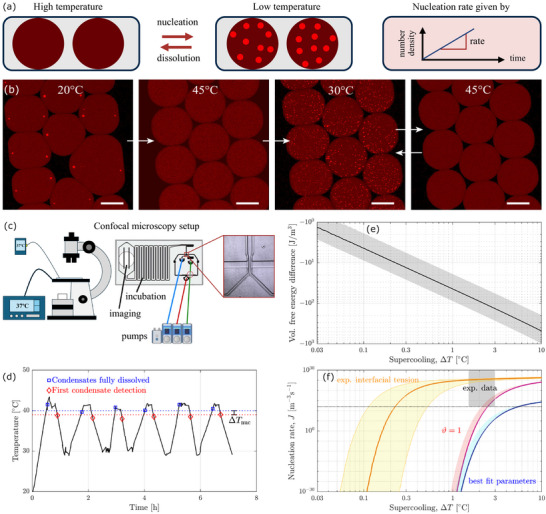
Understanding the nucleation of condensates through the nucleation in condensates. (a) Schematic of the microdroplet assay to measure condensate nucleation rates. (b) Representative images of microdroplets at different temperatures, i.e., before dissolution of the condensates, after dissolution, and after nucleation upon cooling. Scale bar: 100 μm. Note that droplets move due to thermal expansion, therefore the left image shows a different set of droplets than the other images. (c) Schematic of the experimental setup comprising a microfluidic chip loaded with microdroplets on a confocal microscope equipped with a temperature stage. (d) Thermal evolution on the microchip during the experiment comprising six heating–cooling cycles at alternating rates of 0.5 and 1 ${\mathrm{Kmin}}^{-1}$. Dashed lines indicate the highest temperature at which condensates were detected (red) and the lowest where they were fully dissolved (blue). (e) Computed value of the free‐energy difference as a function of supercooling. (f) Nucleation rates as a function of supercooling. The gray rectangle indicates the experimental data, the lines represent the best fit (blue), the fit assuming a correction factor of ϑ=1 (violet), and the prediction using the experimental measurement of γ=0.9 $\mu {\mathrm{Jm}}^{-2}$ (orange). Shades regions represent uncertainty.

**FIGURE 5:**
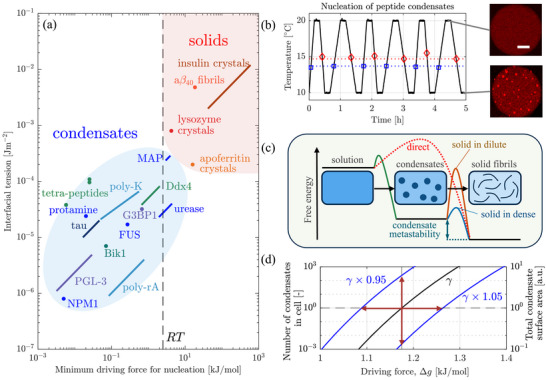
Rapid nucleation enables biological functions of condensates. (a) Nucleation propensity for seventeen compounds (blue – condensates, red – solids) based on their interfacial tensions. Intriguingly, condensates already form at driving forces smaller than RT, whereas solids do not. (b) Experimental measurements of condensate nucleation for the short peptide WGRGRGRGWPGVGYC upon temperature cycling. Both the supercooling at nucleation and the number of condensates that form are similar to what has been observed for poly‐rA RNA condensates. The white scale bar represents 50 μm. (c) Schematic of two pathways for biomolecular liquid–solid transitions: the direct nucleation of solids from solution (red) is slowed down by their large interfacial tension, explaining the role of condensates (green, blue) as metastable intermediates in solidification. (d) Sensitivity study of the number of nuclei forming in a cell as a function of driving force and interfacial tension. Changing γ by 5% (blue vs. black) significantly alters both the number and the surface area of the condensates.

To obtain an experimental reference for the interfacial tension of poly‐rA condensates, we monitored the dynamics of condensate fusion events and computed the capillary velocity (see Figure S4 and Section 4.1 for more details). This velocity is linked to viscosity and interfacial tension, according to the relation $v=\gamma /\eta =2\tau_{\mathrm{fusion}}/\left(d_{1}+d_{2}\right)$, where $\tau_{\mathrm{fusion}}$ denotes the characteristic time of fusion and $d_{1,2}$ the diameters of the two condensates that fuse [50, 51]. The viscosity was obtained from the diffusion coefficient measurements discussed above using the Stokes‐Einstein relation. This method yielded an interfacial tension in the range of $\gamma =0.2-0.9\;\mu J\;m^{-2}$ (red line), depending on the hydrodynamic radius of the poly‐rA RNA, for which we consider values of 5 and 20 nm as physically realistic boundaries given chain lengths in the range of 2000–10000 units [52]. While there is no quantitative agreement, the measured values are of the same order of magnitude as those derived from void nucleation (1.8$\;\mu J\;m^{-2}$), which provides further evidence that the models used here accurately reflect the physics of void nucleation. A plausible reason for the quantitative differences lies in the viscoelastic nature of biomolecular condensates that complicates the relation between fusion velocity and interfacial tension and affects the accuracy of the measurements of γ [5, 53]. This is also supported by the observation of a non‐zero intercept of the diffusion times in the FRAP measurements (see Figure S3) [54, 55].

Finally, a few points are worth noting regarding the simulated evolution profiles; Figure 3c reports for two cooling rates (columns) the profiles of temperature, saturation ratio at r=0 (ratio between actual concentration and the binodal), free‐energy difference at r=0, and nucleation frequency (rows) for three condensates of different sizes (20, 40, 60 μm in diameter, colors). Considering the saturation ratio, the minimum value is attained at the end of the cooling phase, which lies at lower values for larger condensates and faster cooling rates, in line with expectations. Similar trends are observed for the free energy difference, which is more negative, the further the concentration deviates from the equilibrium (i.e., the more the saturation ratio deviates from one); this link between concentration and free‐energy difference is expected, as shown in Figure 2d. With respect to nucleation, larger nucleation frequencies are predicted under conditions where the free‐energy difference is the largest, namely at the end of the cooling phase. After the final temperature has been reached, diffusion homogenizes the concentration across the condensate, and nucleation slows down. If the condensate is sufficiently small (i.e., 20 μm), diffusion prevents the buildup of the driving force required for nucleation to occur, particularly when cooling slowly. This prediction explains the experimental observation that no condensate of <20μm in diameter has experienced nucleation of even a single void. We therefore conclude that the modeling approach followed here accurately describes the thermodynamics and kinetics of void nucleation in condensates. This motivates us to extend the theory to the inverse phenomenon, the biologically relevant nucleation of condensates from a supersaturated solution.

### 2.4 Small Interfacial Tensions Promote the Nucleation of Condensates

Classical nucleation theory has been successfully applied to a wide range of nucleation phenomena, regardless of whether the nucleating phase is a vapor, (dense) liquid, or solid [26, 27, 28, 29]. In the context of phase separation, we utilize the theory to formulate the nucleation rates of both voids and condensates, where the rate of condensate nucleation is given as

(5) $$J_{\mathrm{cond}}=\vartheta D_{-}c\sqrt{\frac{\Delta g_{+}^{2}}{v_{m,+}^{2}\gamma k_{b}T}}\exp\left(-\frac{W_{c,+}}{k_{b}T}\right)$$

and where the rate for voids is given by Equation (3) above. Several points are worth making with respect to $J_{\mathrm{void}}$ and $J_{\mathrm{cond}}$. Both rates are governed by the free‐energy difference $\Delta g_{\pm }$ and the interfacial tension γ: the value of γ is the same regardless of whether a dilute phase is formed within a dense phase or vice versa. The free‐energy difference of condensate nucleation is given as [41, 56]

(6) $$\Delta g_{+}=-k_{b}T\ln\left(S\right)=-k_{b}T\ln\left(\varphi /\varphi_{-}\left(T\right)\right)$$

and the ratio $\Delta g/v_{m}$ in the expressions of J is of the same order of magnitude (≈100 J $m^{-3}$) for both processes under the relevant conditions, as shown in Figures 3c and 4e. This is physically intuitive because in both cases the same two phases are involved and the phase transition merely involves de‐mixing. This similarity results in similar critical nucleus sizes ($r_{c}=2\gamma v_{m}/\left|\Delta g\right|\approx 10-100$ nm) and thus free‐energy barriers of nucleation. Concerning kinetics, diffusion in the condensate is expected to be 2−3 orders of magnitude slower than in the dilute phase, which balances the differences between the concentration levels in the two phases; therefore, the pre‐exponential terms as a whole are expected to be similar for both nucleation phenomena.

The conclusion of this analysis is intriguing: since all terms in the nucleation rate expressions are similar, voids and condensates should nucleate with similar rates. To probe this prediction, we consult again Figure 2b: a close inspection reveals that many small condensates nucleate from the dilute phase upon cooling, in parallel to the voids that nucleate inside large condensates.

This is encouraging from a qualitative perspective, but a quantitative interpretation would be challenging because of the complex interaction between existing condensates and newly formed ones. Thus, we further probed the nucleation of condensates through a different type of experiment. Briefly, we prepared a population of droplets containing poly‐rA RNA in KCl and imaged them under a confocal microscope while changing the temperature (see Section 4.1 for more details), as illustrated in Figure 4a–c. The condensates disappeared after heating and formed in greater numbers when cooled at rates of 0.5 ${\mathrm{Kmin}}^{-1}$ and 1 ${\mathrm{Kmin}}^{-1}$ from about 45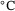 to 30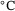, as shown in Figure 4b. Counting condensates after cooling to the minimum temperature of 30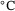 in ten microdroplets resulted in values of 330±60 and 540±70 condensates per nanoliter for the two cooling rates. This information is translated into a nucleation rate assuming that these nuclei form within a timescale of 100s, yielding $J>10^{12}$ $\;m^{-3}s^{-1}$ for both cooling rates. It represents a lower bound of the actual nucleation rate because the growth of the initial nuclei depletes the concentration in the dilute phase, thus slowing down further nucleation events, and because the nuclei may coalesce before the time the image is taken.

To compare this rate with theoretical predictions, we must constrain the range of the driving force in the microdroplets: we measure the temperature difference ΔT between the appearance and disappearance of the nuclei over multiple temperature cycles, finding 1.5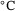 <ΔT< 3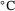. Importantly, the temperatures at which nuclei appear and disappear remained constant over the timescale of the experiment (8 h, see panel (d)), which implies that condensate formation is reversible, as expected from equilibrium. We plot the experimental range of ΔT as a gray rectangle in Figure 4f, which shows the nucleation rates obtained from Equation (5) as a function of the thermodynamic driving force, rescaled in terms of supercooling (see panel (e)). Here, ΔT represents the difference in free energy that arises when a solution of constant composition is cooled from a given temperature (here: 40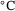).

The nucleation rates predicted using the best‐fit parameters (blue line, uncertainty as shaded region) are about five to ten orders of magnitude lower than the experimental estimate. Given that predictions of nucleation rates span hundreds of orders of magnitude, such a level of agreement is considered rather satisfactory and is routinely observed when applying Classical Nucleation Theory, even in the case of simpler model compounds such as hard‐sphere colloids or crystals of small molecules [57, 58]. Another way to compare experiments and theory is to consider the value of ΔT required to reach the experimentally observed nucleation rate, which is 4.8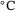 $<\Delta T<5.5^{\circ }C$, i.e., the same order of magnitude as the experimental values.

A potential reason for the under‐prediction of the nucleation rate may lie in the value of the correction factor (best fit $\vartheta =1.5\times 10^{-10}$). In principle, ϑ should be of the order of one for homogeneous nucleation (not catalyzed at interfaces) that is rate‐controlled by volume diffusion [40]. This physical picture may describe well the nucleation of condensates from solution but is potentially less accurate in describing the nucleation of voids, where a large number of RNA molecules must diffuse away from the void – a slow process that may result in a small value of ϑ. We therefore explored how the predicted nucleation rates for condensates change when setting ϑ=1 (violet line); and, indeed, we find a close agreement between experiment and theory with a value of 2.1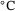 $<\Delta T<2.5^{\circ }C$. Nucleation accelerates further (0.12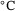 $<\Delta T<0.52^{\circ }C$) when inserting the experimental estimate of the interfacial tension obtained from the fusion experiment (0.9$\;\mu J\;m^{-2}$, orange line). Together, these findings lead to the conclusion that condensates nucleate rapidly because of their low dense–dilute interfacial tension.

Finally, consider the shape of the nucleation rate profiles: J increases strongly at small ΔT, before approaching a plateau; that this plateau region is already reached at low values of ΔT indicates that nucleation proceeds rapidly upon crossing the binodal. This is in line with not only our experiments, but with a wide body of literature where nucleation of condensates is generally perceived as fast, too fast for quantitative studies [15, 59, 60]. This is why we interpret the estimate obtained from the microdroplet assay only as a lower bound of the rate. Instead, we propose both the study of void nucleation and the measurement of the dense–dilute interfacial tension as alternative means to characterize the nucleation of condensates that are experimentally easier to access.

## 3 Discussion and Conclusions

The results presented above establish the dense–dilute interfacial tension γ as the main determinant of condensate nucleation. Measurements of γ are widely available, which allows us to apply Equation (3) to predict the nucleation behavior of thirteen condensate systems. Briefly, we calculate the driving force $\Delta g_{\min}$ at which nuclei form at a relevant rate, namely $J\left(\gamma ,\Delta g_{\min}\right)=10^{12}$ $m^{-3}s^{-1}$ (see Methods for details), and plot it versus γ in Figure 5a. As is evident, small driving forces induce nucleation in all systems, already at values smaller than the thermal energy RT≈2.5 ${\mathrm{kJmol}}^{-1}$. This holds even if the condensate‐forming molecules are very different from poly‐rA RNA, e.g., for intrinsically disordered proteins or for short peptides. We confirm this through an additional case study using a designer peptide with sequence WGRGRGRGWPGVGYC [61] (see Methods), as shown in Figure 5b: when cycling the temperature between 10 and 20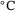, many condensates reversibly form and dissolve at supercooling levels of 1.2 and 1.7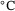 for cooling rates of 0.5 and 1.0 ${\mathrm{Kmin}}^{-1}$, respectively. Together, these findings establish rapid nucleation as a general property of biomolecular condensates and provide a theoretical basis for characteristic features of condensates in biological systems, namely their rapid assembly, high number density, and large surface–volume ratio.

These results rely on Classical Nucleation Theory (CNT), which describes nucleation as a single‐step process. In this context, it is worth mentioning that mesoscale clusters with sizes of hundreds of nanometers have been observed for several condensate‐forming proteins, including FUS [62], A1‐LCD [17], and CPEB4 [63]. Such clusters may affect nucleation in two ways: first, they may be off‐pathway and act as sinks that deplete the monomer concentration, lower the driving force, and thus slow down nucleation. According to the literature, the cluster population contains less than one percent of the biomolecule (for FUS [62]), making this effect insignificant. Second, they may be on‐pathway and act as seeds that grow into condensates as soon as the system becomes supersaturated. In this case, the total nucleation rate would be a sum of the contributions from the clusters and from the single‐step pathway; that is, nucleation is even more rapid as predicted in Figure 5a, although the extent of this effect is currently not yet known. For subsaturated poly‐rA RNA in 1 M KCl, DLS measurements (Methods, Figure S7) reveal a cluster size distribution with a single peak at a diameter of about 40 nm at 55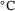 and 60 nm at 40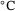. The diameter of a single poly‐rA RNA molecule lies between 10 and 40 nm depending on its chain length. This confirms that RNA forms oligomeric clusters in solution, although to a lesser extent than the aforementioned condensate‐forming proteins. The larger diameter at lower temperature is consistent with the observed upper critical solution behavior (see Figure 2c): at a given RNA concentration, cooling shifts the subsaturated solution closer to saturation, which increases its tendency to form oligomers (and thus the size seen in DLS), as is expected from equilibrium thermodynamics. For the poly‐rA RNA condensates studied in this work, the measured nucleation rate agrees closely with that predicted by CNT, which supports the notion that the single‐step mechanism is prevailing. An exciting avenue for future studies would be to elucidate the nucleation kinetics of condensates made up of proteins that are known to form mesoscale clusters: since void nucleation takes place in a single step, its study provides a reliable estimate of the interfacial tension that allows predicting single‐step condensate nucleation rates, as we did here for poly‐rA RNA. Comparing this estimate with direct measurements of condensate nucleation rates then enables us to assess whether mesoscale clusters substantially affect nucleation rates.

Regardless of whether condensates nucleate in a single step or in multiple steps via mesoscale clusters, their rapid nucleation distinguishes them from solids: we computed the driving force for the nucleation of four solids (three protein crystals and amyloid $\beta_{40}$) using the same approach and found that greater driving forces are required to induce their nucleation due to higher interfacial tensions. This agrees well with the notion that biomolecules crystallize and aggregate rather slowly: aggregates form mainly through secondary processes with only a single or a few primary nucleation events per cell [64, 65], in stark contrast to the rapid assembly of numerous condensates upon stress [15, 19, 20]. Thus, the distinct nucleation behavior of condensates and solids results in unique biological function spaces.

This distinction further implies that, in the presence of a driving force, condensates may nucleate before solids. As shown in Figure 5c, solids can form directly from a supersaturated solution or through condensates as an intermediate. The direct pathway is unfavorable due to high interfacial tensions, which favors widely reported two‐step pathways [9, 24, 66, 67]: condensates appear first and promote the nucleation of solids because their high internal concentration lowers the interfacial tension. For insulin crystals [68], a solid–dense tension of 0.2 mJ ^−2^ and a solid–dilute one of 2–12 mJ $m^{-2}$ were reported. Given typical levels of metastability of condensates of 1–10 kJ mol^−1^ [66, 67, 69], this decrease in interfacial tension may accelerate nucleation to a biologically relevant level. Alternatively, condensates represent a kinetically‐arrested state and act as sinks that prevent further solidification, a behavior that has been observed experimentally as well [69, 70].

Finally, we note that γ provides a straightforward pathway for modulation. Since its value is sensitive to the composition of the solution (see e.g., the review of Zhou et al. [5]), cells can control the rate of condensate nucleation by modulating their composition. Intriguingly, changing γ only by a few percent changes the number of nuclei formed in a cell by orders of magnitude – see Figure 5d. Importantly, such a change in γ directly affects the total surface area of the condensates and therefore represents an avenue for altering the rate of surface‐active processes such as redox reactions [23], enzymatic reactions [25], or protein aggregation [9, 24].

## 4 Materials and Methods

### 4.1 Experimental Methods

#### 4.1.1 Materials

Poly‐rA (700‐3500 kDa, lyophilized powder, 10108626001) and KCl were obtained from Sigma–Aldrich. U80‐Cy5 RNA and U20‐Cy5 RNA for fluorescent labeling were purchased from GenScript. 1M Tris buffer at pH = 7.5 was obtained from Thermo Fisher.

#### 4.1.2 Experimental Setup

A Leica Stellaris 5 confocal microscope (white light laser) microscope with a 10x air Leica PlanFluor objective was used for imaging. The temperature was controlled using a TS102SI Instec rapid heating and cooling stage with built‐in software and measured using a K‐type thin‐wire thermocouple with exposed tip that was installed on the chip. Analysis was performed using Fiji (2.3.051) [71], Origin (2017), and Adobe Illustrator (29.3.1).

#### 4.1.3 Binodal Concentrations

The dilute phase poly‐rA concentrations were estimated by incubating samples for 10 min and spinning at 21000 g for 15 min at the specified temperature and then taking a NanoDrop measurement of the supernatant (NanoPhotometer NP80, Geneflow). Dense phase concentrations were estimated using the confocal microscope described above in photon count mode. At constant excitation power, the photon counts in homogeneous samples with known concentration levels of Cy5 were compared to the dense phase counts to estimate the concentration of RNA in the condensates. This approach was described in more detail in earlier work [30].

#### 4.1.4 Diffusion Coefficient

Fluorescence recovery after photo‐bleaching (FRAP) measurements (Figure S3) were carried out at 45.6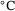 following the method described in the literature [55, 72]. Specifically, the Leica Stellaris  confocal microscope was used to bleach circular areas of various sizes into condensates. The fluorescent intensity over time, I(t), was fitted using $I\left(t\right)=I_{\mathrm{final}}-\left(I_{\mathrm{final}}-I_{\mathrm{start}}\right)e^{-t/\tau }$ with the Leica built‐in software (Figure S3b), yielding τ, the characteristic time of diffusion into the bleached area. Using the diameter of the bleached areas, d, and τ, we find the diffusion coefficient using $D=d^{2}/\left(16\tau \right)$ [55]. Bleaching was performed at 100 % laser power at 647 nm.

#### 4.1.5 Void Nucleation

Samples for void nucleation experiments were prepared that contained 5 g L^−1^ poly‐rA, 0.05 g L^−1^ U80‐Cy5, 1 M KCl and 50 mM tris pH = 7.5. Samples were stored in glass and PDMS sealed wells. We refer to earlier work for a detailed description of the cooling experiments [30, 31].

#### 4.1.6 Condensate Nucleation

Condensate nucleation experiments were performed using the confocal microscope and the temperature stage described above. Microfluidic devices to generate microdroplets were prepared according to standard soft lithography practices based on designs used previously [73]. Stable droplets were obtained using three pressure‐controlled pumps (LineUp Flow EZ, Fluigent) injecting at 100 µL h^−1^ each (i) FC‐40 oil with 10% (w/v) fluorosurfactant as continuous phase, (ii) aqueous 2M KCl solution with 1 µM Alexa Fluor 488, and (iii) aqueous 1.64 g L^−1^ poly‐rA solution with 1% U20‐Cy5. The use of two aqueous streams instead of a single pre‐mixed one provides several practical benefits: it lowers the variability in composition among droplets and avoids clogging of inlets and droplet junction. The addition of Alexa Fluor 488 dye facilitates the detection of droplets on the confocal microscope. Microdroplets containing 0.82 g L^−1^ poly‐rA, 1 M KCl, 0.0082 g L^−1^ U20‐Cy5, and 0.5 µM Alexa Fluor 488 were collected on a microchip and transported to the confocal microscope to undergo temperature change experiments. The outlets of the chip were sealed using glue to slow down evaporation; no relevant evaporation was observed during the experiments that took up to 8 h. The temperature was measured using a thin wire K‐type thermocouple inserted into the chip. Six heating–cooling cycles were carried out per experiment at alternating rates of 0.5 and 1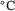 min^−1^. The experiment was carried out three times to ensure reproducibility.

Additional nucleation experiments were performed for the peptide WGRGRGRGWPGVGYC, which was obtained as a lyophilized powder from GenScript Biotech (UK). This sequence (without C) was reported to undergo phase separation under a wide range of conditions and therefore considered a suitable peptide model system [61]. It is enriched in arginine and glycine residues, which facilitate electrostatic and cation–π interactions that favor phase separation. The peptide was dissolved in 50 mM Tris buffer at a final concentration of 4.41 g L^−1^. The samples were supplemented with 1 μ M Alexa Fluor 488 and 1 μ M Alexa Fluor 647 for labeling, allowing condensate detection thanks to the selective recruitment of the 647 dye into condensates. 2 M stock solution of Potassium chloride was used. Mixing the two aqueous streams at a 50:80 ratio resulted in a peptide concentration of 1.69 g L^−1^ and 1.2 M KCl.

#### 4.1.7 Condensate Detection

To detect condensate formation and dissolution, Z‐stack imaging (2.4 μm per slice) of the microdroplets was performed continuously throughout the thermal cycles. The time interval between consecutive z‐stack acquisitions was set to the system's minimum, allowing for continuous imaging. The time points of condensate formation and dissolution were identified by manually analyzing the Z‐stacks. To count the condensates, Z‐stacks were analyzed using FIJI's (ImageJ) Analyze Particles tool. Ten droplets were manually selected in each experiment, at a time ten minutes after reaching the minimum temperature of the cycle to ensure thermal equilibrium.

#### 4.1.8 Condensate Fusion

Condensate fusion measurements were performed using a Leica Stellaris 5 confocal microscope and a VaHeat heatstage with smart substrate (600 µL, 5x5 mm^2^, 1.5H) with reservoir cap (5×5 ${\mathrm{mm}}^{2}$) (Interherence) at 52.5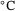. Condensates were imaged over time at 0.52 or 0.371 seconds per frame. The aspect ratio and time were fitted according to

(7) $$A\left(t\right)=1+\left(A_{0}-1\right)\exp\left(-\frac{\tau_{\mathrm{fusion}}}{t}\right)$$

to obtain the characteristic fusion time $\tau_{\mathrm{fusion}}$ [74]. Example images of fusing condensates as well as the fit of the aspect ratio are shown in Figure S4. 12 $\tau_{\mathrm{fusion}}$ values were obtained for condensates of various sizes to determine the capillary velocity.

#### 4.1.9 DLS Measurements

A stock solution that contained 0.1 g L^−1^ poly‐rA RNA and 1 M KCL was prepared and filtered using a 0.2 μm syringe filter. Samples were centrifuged for 15 min at 10,000 g, after which the supernatant was measured. DLS was performed using disposable polystyrene cuvettes with the Litesizer 500 (Anton Paar) and build‐in software. Measurements were performed at 40 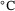 and 55 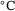 and were repeated six times per temperature.

### 4.2 Flory‐Huggins Fitting

Binodal concentrations at each temperature were calculated using the convex hull algorithm [34, 37], a numerical implementation of Gibbs' graphical construction approach [38, 39]. Volume fractions were converted into mass concentrations assuming an RNA density of 1.6 g mL^−1^ [36]. The dependence of the Flory interaction parameter on temperature was described as χ(T)=A+B/T, where A quantifies temperature‐independent entropic contributions and B the effective pairwise energy originating from the interplay of polymer–solvent, polymer–polymer, and solvent–solvent interactions [33, 35, 75]. Note that both entropic and enthalpic effects are widely considered to be relevant drivers of phase separation [76, 77]. The two parameters were estimated from experimental data. The chain length was set to M=2000, which reflects the shorter end of the chain length distribution of the polydisperse RNA used in this work (between 2000 and 1000 units). Higher values of M were considered, but found to result in an over‐prediction of the effect of temperature on the dilute phase concentration. Similarly, lower values of M resulted in an over‐prediction of the concentration at the critical temperature. Suitable values of A and B were identified by fitting dense phase concentrations to their experimental counterparts, resulting in values of A=0.23 and B=98.8K. To estimate the uncertainty associated with the choice of M and their effect on the subsequent estimation of the interfacial tension, the interaction parameters were fitted to M=1000 (A=0.31 and B=75.0K) and M=4000 (A=0.19 and B=110K) as well. The corresponding fits to the experimental data are shown in Figure S6.

### 4.3 Nucleation Rate Expression

We derive the expressions for the condensate and void nucleation rates from Classical Nucleation Theory (CNT), following the approach reported in the literature for crystal nucleation from solution [40, 41]. According to the theory, nucleation occurs through the attachment and detachment of molecules to molecular clusters. Because both the dense and dilute phases are liquids that mostly consist of water, the effects of compressibility on the nucleation rate are insignificant and therefore are not considered. The nucleation rate is formulated for stationary conditions where the cluster concentrations are constant over time, as the product of three terms

(8) $$J=zf_{c}\rho_{c,m}=zf_{c}\rho_{m}\exp\left(-\frac{W_{c}}{k_{b}T}\right)$$

where z denotes the Zeldovich factor, $f_{c}$ the monomer attachment frequency of critically‐sized clusters, and $\rho_{c,m}$ the number concentration of critically‐sized clusters. $\rho_{c,m}$ is expected to follow a Boltzmann‐type formula and can be written using the number concentration of nucleation sites, termed $\rho_{m}$, for which $\rho_{m}=1/v_{m}$ is commonly assumed in homogeneous nucleation. The Zeldovich factor corrects for the fact that the size distribution of (near) critical clusters in steady‐state nucleation was not equal to the Boltzmann distribution. It is defined as

(9) $$z=\sqrt{\frac{-d^{2}W/dn^{2}{|}_{n=n_{c}}}{2\pi k_{b}T}}=\frac{\Delta g^{2}}{8\pi v_{m}\gamma^{3/2}\sqrt{k_{b}T}}$$

The derivation of the monomer attachment frequency depends on which mechanism is rate‐limiting. Here, we assume that attachment is governed by volume diffusion, in contrast to surface integration, because the formation of voids (and of condensates) represents primarily a density transition. Thus, $f_{c}$ is

(10) $$f_{c}={\left(48\pi^{2}v_{m}\right)}^{1/3}cDn_{c}^{1/3}=8\pi v_{m}cD\frac{\gamma v_{m}}{\left|\Delta g\right|}$$

where c is the number concentration of the polymer in the existing phase. Combining these expressions results in

(11) $$J=\vartheta Dc\sqrt{\frac{\Delta g^{2}}{v_{m}^{2}\gamma k_{b}T}}\exp\left(-\frac{W_{c}}{k_{b}T}\right)$$

whereby a correction factor ϑ has been added to the prefactor to facilitate the quantitative fitting of the nucleation rate from experimental data. This expression applies to both the nucleation of voids from the dense phase and to the nucleation of condensates from the dilute phase. To clarify this, we rewrite the equation using indices to indicate which quantities refer to the dense (+) and dilute (‐) phase, respectively:

(12) $$J_{\mathrm{void}}=\vartheta D_{+}c\sqrt{\frac{\Delta g_{-}^{2}}{v_{m,-}^{2}\gamma k_{b}T}}\exp\left(-\frac{W_{c,-}}{k_{b}T}\right)$$

(13) $$J_{\mathrm{cond}}=\vartheta D_{-}c\sqrt{\frac{\Delta g_{+}^{2}}{v_{m,+}^{2}\gamma k_{b}T}}\exp\left(-\frac{W_{c,+}}{k_{b}T}\right)$$

where the work of forming a critical cluster is

(14) $$W_{c,-}=\frac{16\pi }{3}\frac{\gamma^{3}v_{m,-}^{2}}{\Delta g_{-}^{2}},\;\;\;\;\;\;W_{c,+}=\frac{16\pi }{3}\frac{\gamma^{3}v_{m,+}^{2}}{\Delta g_{+}^{2}}$$

Let us next consider the free‐energy difference. For $\Delta g_{+}$, we rely on Equation (6). $\Delta g_{-}$ is given by Equation (2) and the partition factor K is obtained from a volume balance such that the number of solvent and RNA molecules in the control volume remains constant throughout phase separation:

(15) $$\left(n_{-}+n_{+}\right)\frac{Mv_{1}}{\varphi }=n_{-}\frac{Mv_{1}}{\varphi_{-}}+n_{+}\frac{Mv_{1}}{\varphi_{+}}$$

(16) $$K\left(\varphi ,\varphi_{\pm }\right)=\frac{n_{+}}{n_{-}}=\frac{1/\varphi -1/\varphi_{-}}{1/\varphi_{+}-1/\varphi }$$

where $Mv_{1}/\varphi$ denotes the volume of phase occupied by one RNA molecule and the associated solvent.

### 4.4 Diffusion–Nucleation Model

We simulate the evolution of a single spherical condensate of time‐dependent radius R(t) surrounded by a dilute phase during a cooling process. Given the small size of the condensate in the micro‐meter range, heat transfer is rapid and no relevant temperature gradients are expected along the spherical coordinate r∈[0,R(t)] during cooling, i.e., T(r,t)=T(t). The change in temperature shifts the binodal concentrations such that the condensate becomes undersaturated with respect to the biomolecule. The concentration at the condensate interface is equal to the equilibrium value at the given temperature, i.e., $c_{\mathrm{cond}}\left(R\left(t\right),t\right)=c_{\mathrm{cond}}^{*}\left(T\right)$. Due to the low diffusion coefficient on the order of 0.1 $\;\mu m^{2}\;s^{-1}$, mass transfer was sufficiently slow to allow for the formation of concentration gradients:

(17) $$\frac{\partial c_{\mathrm{cond}}}{\partial t}=D\frac{1}{r^{2}}\frac{\partial }{\partial r}\left(r^{2}\frac{\partial c_{\mathrm{cond}}}{\partial r}\right)$$

At the boundary, r=R(t), we assume that no exchange of the polymer between the condensate and the dilute phase takes place. Accordingly, the mass of the polymer in the condensate was conserved, so that

(18) $$\int_{0}^{R(t)}4\pi r^{2}c_{\mathrm{cond}}\left(r,t\right)dr=\mathrm{constant}$$

which provides an implicit relation for the evolution of R(t): as the condensate becomes more dense upon cooling, it shrinks in size. The initial state of the condensate at time $t=t_{0}$ is given by a uniform temperature $T\left(t_{0}\right)=T_{0}$ and solute concentration $c\left(r,t_{0}\right)=c_{\mathrm{cond},0}$, and a radius $R\left(t_{0}\right)=R_{0}$. The temperature was assumed to decrease at a constant rate α according to $T\left(t\right)=T_{0}-\alpha t$ down to a value of $T_{\mathrm{end}}$. The simulations were completed at time $t_{\mathrm{end}}$ when the condensate concentration was equilibrated at the final temperature.

The evolution of concentration and condensate size was obtained numerically using a finite difference approach [78] with a 1D spherical geometry that had been implemented in MATLAB R2022b. Concerning size evolution, the condensate was assumed to shrink in finite steps, in line with the chosen discretization: in every time step, the mass of polymer in the condensate was computed considering all but the outermost grid point. If this mass was larger than the initial mass, the outermost grid point was removed and the condensate shrinks accordingly.

### 4.5 Nucleation Parameter Estimation

The parameters γ and ϑ in the nucleation rate expression (Equations (3) and (5)) were estimated by fitting simulations of the nucleation–diffusion model to the measured critical condensate sizes where N=1. Three different expressions for γ were considered, a temperature‐independent one, and two scaling laws. Physically, we aim to find the set of parameters for which the number of voids that form in critically‐sized condensates at all cooling rates was as close to one as possible. Mathematically, the best‐fit parameters were identified by minimizing the objective function

(19) $$\min_{\gamma_{0},\vartheta }\sum_{k}\ln{\left(N\left(R_{0,k}\right)\right)}^{2}$$

whereby we use the logarithm because it holds that ln(1)=0 and it gives similar weights to simulations that under‐ and over‐predict the number of voids. To ensure that the identified parameters correspond to the global minimum, the solver was initialized with a wide range of initial guesses.

The nucleation–diffusion model predicts the concentration field inside a condensate of a given size. This allows calculating the expected number of voids that nucleate (see Equation (4)), for a given set of nucleation parameters. The optimal parameters were defined such that the simulated number of voids was as close to one as possible at all cooling rates. They were identified by minimizing the objective function given by Equation (19) using the Nelder‐Mead algorithm [79]. To assess the uncertainty of this approach, parameters were estimated using three different parameterizations of the Flory‐Huggins model, as outlined above.

Having identified the best‐fit parameters, these parameters were used to calculate the critical sizes at all cooling rates by identifying the size for which N=1. This was achieved using a bisection method starting with a very small (d=10μm, N<<1) and a very large size (d=100μm, where N>>1) as limits, using that N increases monotonously with d. To account for the stochasticity of nucleation, we additionally calculated the sizes where N=0.242 and N=5.572 (see Section S.1).

Reference simulations were carried out using condensates of three sizes (d=20μm, d=40μm, d=60μm) at the two cooling rates that correspond to the fastest and slowest rates observed in the experiments. The nucleation frequency K was computed by integrating the nucleation rate over the condensate volume.

### 4.6 Condensate Nucleation Prediction

Condensate nucleation rates were predicted using Equation (5). We relied on the parameters estimated for void nucleation using the Ising temperature‐scaling for γ; for predictions that rely on the interfacial tension from fusion experiments, a constant value of 0.9$\;\mu J\;m^{-2}$ was used. The diffusion coefficient in the dilute phase was set to $D_{-}=1000\times D_{+}$ and the concentration in the pre‐factor was set to the experimental value of 0.82 mg mL^−1^. The supersaturation was computed using the dilute phase binodal as predicted by the Flory‐Huggins model with the value at 40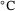 as a reference. Supercooling was defined as the difference between the actual temperature and 40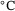. A temperature resolution of 0.003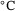 was used.

To assess the uncertainty of these predictions, they were carried out using three different parameterizations of the Flory‐Huggins model. This results in an uncertainty in the value of the driving force that propagates to the nucleation rates. Because this uncertainty also affects the estimates of the interfacial tension in the void nucleation approach (greater driving force results in higher interfacial tension), it had only a small effect on the corresponding predictions of condensate nucleation. However, when the interfacial tension of the fusion experiments was used instead, the resulting uncertainty was greater.

### 4.7 Analysis of Interfacial Tension Data

Interfacial tension values for various condensate systems were collected from the literature: for NPM1 [7], PGL‐3 [80], Bik1 [81], urease [82], Ddx4 [83], protamine [84], poly‐K [84], three tetra‐peptides [85], tau [86], FUS [87], G3PB1 [87], and MAP [88]. For poly‐rA RNA, the values measured in this work were used. When selecting literature values, we focused on condensates containing only a single active biomolecule and measurements with reasonable experimental errors. Where multiple estimates were available, for example, because the interfacial tension was measured for different solution compositions, the lowest and highest values of γ were taken.

Application of Equation (3) to these literature values requires additional information. We obtained molecular weights from the literature, accounting for oligomeric states of the biomolecules, and computed the volume $v_{m}$, assuming a volume fraction of 0.2 for all condensates. The mass concentration was set to 1 mg mL^−1^, the temperature to 300 K, the solute density to 1.5 g mL^−1^, and the diffusivity to $10^{-10}$ $\;\;m^{2}\;s^{-1}$. Although the actual values of these quantities in practice differ between individual condensate systems, their effect on the overall analysis is minor compared to that of the interfacial tension, which justifies the use of a single set of values. Interfacial tensions of solids were taken from the literature as well: for insulin crystals [68], lysozyme crystals [89], amyloid $\beta_{40}$ fibrils [90], and apoferritin crystals [91], and a volume fraction of 0.5 was used in all calculations.

The number of nuclei that form in a cell, as shown in Figure 5d, was calculated using a modified version of Equation (4): we consider a characteristic time between the formation of the driving force and its depletion via diffusive transport as $\Delta t=d^{2}/\left(6D\right)$ where D is the diffusion coefficient of the solute in the cell and d=60μm the diameter of the cell assuming spherical geometry. N is computed as N=J(ΔG,γ)VΔt where V denotes the volume of the cell. A value of γ=200μJ $m^{-2}$ was used, reflecting the upper limit of what was observed for condensate systems. N was calculated as a function of Δg for this value and for values 5% higher and lower to assess its sensitivity. The relative surface area of the condensates scales with a third of the power of the number of condensates, e.g., if at a given condition 1000 times more condensates form but with the same total mass, then their diameter is ten times smaller, and thus their total surface area is ten times larger.

### 4.8 Statistical Analysis

**Void Nucleation**. Both size and number of voids were measured for a large number of condensates (>50) per cooling rate. The error bars represent two standard deviations of the mean size; all data points were included in the analysis, except for two points where the sizes deviated more than two standard deviations from the mean. The uncertainty in the fit of the critical sizes was obtained from a stochastic description of nucleation (see Section S.1 for details). The uncertainty in the estimate of the interfacial tension was computed from the uncertainty in the binodals (see Section 4.2 for details). **Condensate Nucleation**. Temperature‐cycling experiments for poly‐rA RNA were carried out three times to ensure reproducibility; the peptide experiment was carried out once. Nucleation rates were averaged over ten droplets. Detection and melting temperatures of condensates were obtained for each temperature cycle (six cycles per experiment). **Diffusion Coefficient**. The diffusion coefficient was obtained by fitting the equation of the characteristic time to nine data points that correspond to bleached areas of different sizes. **Condensate Fusion**. Twelve condensate fusion events were monitored to obtain the estimate of the capillary velocity.

## Nomenclature

**Symbols**
M
: effective chain length −
N
: number of nuclei −
φ
: solute volume fraction −
χ
: Flory interaction parameter −
W
: work of cluster formation J
γ
: interfacial tension J $m^{-2}$
n
: number of solute molecules −
Δg
: free energy difference J
f
: free energy per lattice site J
$v_{1}$
: volume per lattice site $m^{3}$
K
: partition factor −
$v_{m}$
: expected volume per solute molecule $m^{3}$
T
: temperature 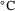

ΔT
: supercooling 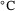

ϑ
: pre‐factor −
c
: number concentration $m^{-3}$
J
: nucleation rate $m^{-3}$ $s^{-1}$
$k_{b}$
: Boltzmann constant J $K^{-1}$
$N_{A}$
: Avogadro constant ${\mathrm{mol}}^{-1}$
$M_{w}$
: Molecular weight constant kg ${\mathrm{mol}}^{-1}$
$\hat{c}$
: mass concentration kg $m^{-3}$
D
: $\mathrm{diffusivity}\;m^{2}$ $s^{-1}$
r,d
: condensate radius, diameter m
$R_{0,k}$
: critical radius for cooling rate k m
v
: capillary velocity m $s^{-1}$
η
: viscosity Pa s
$\tau_{\mathrm{fusion}}$
: characteristic time of condensate fusion s

**Subscript**
c
: critical
±
: dense (+) or dilute (‐) phase
void: void
cond: condensate
0,1,2: multiple uses

## Author Contributions

L.T.D. and N.A.E conceived the study. L.T.D. developed the theory and carried out the simulations. N.A.E., S.N.P, and L.T.D. carried out the experiments. L.T.D. and N.A.E. analyzed the results. L.T.D. wrote the manuscript with the support of all authors. T.P.J.K. supervised the study.

## Conflicts of Interest

T.P.J.K. is the co‐founder of Fluidic Analytics, Wren Therapeutics, Xampla, and Transition Bio. These associations did not influence the work reported here. The remaining authors declare no conflicts of interest and none of the authors have any non‐financial interests to declare.

## Data Availability

All data and code generated or analyzed during this study are included in this article and its supplementary figures or are available upon request by contacting the corresponding authors: n.a.erkamp@tue.nl (N.A.E.) and tpjk2@cam.ac.uk (T.P.J.K.).
